# Comparative Efficacy of Levetiracetam for Epilepsy in School-Aged Children with Intellectual Disability and Normal Intelligence

**DOI:** 10.3390/brainsci11111452

**Published:** 2021-10-31

**Authors:** Ja Un Moon, Ji Yoon Han

**Affiliations:** 1 Department of Pediatrics, College of Medicine, The Catholic University of Korea, Seoul 06591, Korea; gonicky@naver.com; 2 Department of Pediatrics, Seoul St. Mary’s Hospital, The Catholic University of Korea, Seoul 06591, Korea; 3 Department of Pediatrics, Daejeon St. Mary’s Hospital, The Catholic University of Korea, Daejeon 34943, Korea

**Keywords:** levetiracetam, anti-seizure medications, intellectual disability, epilepsy, children, efficacy, adverse reaction, retention rate, monotherapy, polytherapy

## Abstract

Choosing optimal anti-seizure medication (ASM) is very important in pediatric patients with epilepsy who attend school, especially children with an intellectual disability (ID). Levetiracetam (LEV) has proven to be an effective, safe, generally well-tolerated, broad-spectrum ASM in children. In the context of increasing use of LEV in school-aged children with epilepsy and ID, we evaluate relevant clinical data, including efficacy, safety, and tolerability in children with epilepsy and an intellectual disability (ID) or normal intelligence (NI). We performed a retrospective chart review of children and included 298 pediatric patients with epilepsy who were treated with LEV with NI (147) and ID (151). After 6 months, 96% of NI and 83% of ID subjects had a seizure reduction rate greater than 50% (*p =* 0.031). The tolerability of LEV was generally good, with 75% retention rates at 2 years in both groups and only minor side effects (under 15%). The retention rates of patients with NI and ID were 76% and 74%, respectively (*p =* 0.597). Thus, LEV showed considerable efficacy with minimal side effects and high retention rates and is an easily maintained and safe treatment option for pediatric epilepsy with ID. However, better-designed research studies are needed to clearly elucidate the efficacy and safety of LEV in children with epilepsy and ID.

## 1. Introduction

Epilepsy is one of the most common major neurological diseases in the pediatric population, affecting 4–10 per 1000 children [1]. Neurodevelopmental disorders, including developmental delay (DD) or intellectual disability (ID), are common in pediatric patients with epilepsy and are likely to increase the risk of seizures [2]. The prevalence of epilepsy in children with ID ranges from 30 to 50%, which is higher than in the general population and tends to be poorly controlled [3]. In addition, children with ID are more likely to develop intractable epilepsies and might experience further intellectual deterioration resulting from inappropriate use of anti-seizure medication (ASM). However, some children with ID experienced improvement of cognitive ability after seizure control [2]. Given these factors, treatment strategies should focus on controlling seizures and managing behavioral and cognitive comorbidities. Unfortunately, the treatment of choice for epilepsy in children with DD/ID has not been well-established, and guidelines for ASM for children with ID are not different from those of children with solitary epilepsy. Therefore, more evidence-based information on treatment options is needed to address the insufficiency of current treatment strategies.

Levetiracetam (LEV) has been proven to be an effective, safe, generally well-tolerated, and broad-spectrum ASM in children with focal or generalized epilepsy [4]. LEV has favorable benefits that make it effective and suitable for children with epilepsy: optimal bioavailability, no interactions with other ASMs, low protein binding, and multiple drug formulations [5]. In addition, LEV is important for children during development and learning periods as it positively affects behavior and cognitive functions [6]. However, despite the increasing use of LEV in school-aged children with epilepsy and ID, data on the efficacy and safety of LEV for such children remain insufficient. Therefore, this study aims to evaluate the efficacy, tolerability, and safety of LEV in school-aged pediatric patients with epilepsy and ID by comparing this population to children with normal intelligence (NI).

## 2. Methods

We performed a retrospective chart review of children aged 6–18 years with epilepsy and ID who visited The Catholic University of Korea Seoul St. Mary’s Hospital between January 2010 and May 2019. Children were eligible for inclusion if they were treated with LEV and followed for more than 2 years. One pediatric neurologist reviewed the medical charts of all patients and extracted information including demographics (age, gender), seizure type, clinical findings, brain magnetic resonance images (MRI), biochemical results, and concomitant ASM. The mean LEV dosage, duration of LEV treatment, and dropout rate of LEV were assessed after 6, 12, and 24 months. Outcomes of seizures following LEV treatment were assessed in the first 6 months and defined based on seizure frequency or clinical manifestations as no change or <50% reduction in seizure frequency, 50–99% reduction in seizure frequency, and seizure-free. To determine the sole effect of LEV in polytherapy, the frequency of seizures of each group before and after LEV addition was also evaluated. Safety assessments, including adverse reactions, laboratory parameters, vital signs, physical and neurological examinations, and electrocardiography, were conducted over 6, 12, and 24 months. The definition of epilepsy and the classification of seizures were determined according to categories proposed by the International League Against Epilepsy (ILAE) in 2014 and 2017, respectively [7,8]. Intelligence quotient (IQ) of 70 or below with at least two behaviors related to adaptive functioning deficits manifesting before age 18 was defined as ID. The levels of severity of ID were classified into mild (IQ of 50–69), moderate (35–49), severe (20–34), and profound (<20) by the Wechsler intelligence test or Wechsler Preschool and Primary Scale of Intelligence. We performed all statistical analyses using SSPS software version 24.0 (IBM Corp., Armonk, NY, USA), and continuous variables were presented as means and standard deviation (SD). The relationship between the before and after the addition of LEV in polytherapy was evaluated using a paired t-test. We compared categorical variables using Chi-square/Fisher’s exact test and assessed continuous variables using Kruskal–Wallis test to analyze significant differences between two groups (NI and ID). We considered probability values less than 0.05 as statistically significant. This study was approved by the Institutional Review Board of The Catholic University of Korea (KC21RASI0633).

## 3. Results

### 3.1. Demographics and Clinical Characteristics

A total of 298 pediatric patients with epilepsy who were treated with LEV was included in this study. The demographic and clinical characteristics of the patients are shown in Table 1. Among the 298 patients, 147 (70 females, 77 males) had NI, and 151 (71 females, 80 males) had ID. Of 151 patients with ID, 74 (49%) had mild ID, 54 (36%) had moderate ID, and 23 (15%) had severe to profound ID. The mean ages of patients with NI and ID were 12.3 and 12.0 years, respectively. The mean age at onset of seizures in NI was 5.5 years (range: 1 month–15 years) and in ID was 4.5 years (range: 1 week–15 years). In patients with NI, 28 (19%) had localized seizures, and 83 (56%) had generalized seizures, while the rest (*n* = 36, 25%) had both.

Among ID patients, 17 (11%) had localized seizures, 81 (54%) had generalized seizures, and 53 (35%) had both types. Most patients with NI had unknown causes of epilepsy (*n* = 104, 71%) followed by genetic causes (*n* = 10, 7%) such as Prader–Willi syndrome, Fragile X syndrome, or microdeletion/duplication, but not all patients had undergone genetic testing. Structural malformation (e.g., lissencephaly, cortical dysplasia) and perinatal problems (e.g., hypoxic insult) accounted for a large proportion of the causes of epilepsy in ID patients (*n* = 43, 29% and *n* = 30, 20%, respectively) (*p* = 0.044). There was no statistically significant difference between the two groups in gender, mean age, mean age of seizure onset, seizure type, or etiology (*p =* 0.918, 0.422, 0.232, 0.390, and 0.074, respectively). Brain MRI analyses showed abnormalities to be significantly more common in ID patients (*n* = 99, 66%) compared to patients with NI (*n* = 44, 30%) (*p* < 0.044). The mean dosage of LEV per day was 33 mg/kg in both groups, ranging from 10 mg/kg to 60 mg/kg (*p =* 0.517). Significantly more patients with NI (*n* = 51, 35%) had received LEV as a single treatment (monotherapy) compared to those with ID (*n* = 36, 24%), while more patients with ID (*n* = 115, 76%) received LEV as a combined treatment with other ASMs (polytherapy) than those with NI (*n* = 96, 65%) (*p =* 0.047). Among the polytherapy group, patients with ID required more ASMs in addition to LEV treatment: one (*n* = 68, 70%), two (*n* = 21, 22%), or more than two (*n* = 7, 8%) in patients with NI, whereas patients with ID showed one (*n* = 50, 44%), two (*n* = 36, 31%), and more than two (*n* = 29, 25%) additional ASMs.

### 3.2. Adverse Effects

Overall, there were no significant differences in the adverse reactions between patients with NI and ID (*p =* 0.397) (Table 2) or between monotherapy and polytherapy (*p =* 0.411). A total of 35 (12%) of all patients (20 with NI, 15 with ID) experienced adverse reactions, and none had life-threatening events. Most patients experienced adverse reactions within three months of beginning LEV treatment, resulting in immediate treatment interruption in some cases. However, no such differences were associated with adverse reactions and a mean dosage of LEV (*p =* 0.377) in all patients. The most frequently observed adverse reactions were sedation (*n* = 17, 49%), including fatigue and somnolence, followed by neuropsychiatric events (*n* = 8, 23%), including aggression/irritability and depression, headaches (*n* = 5, 14%), dizziness (*n* = 4, 11%), and loss of appetite (*n* = 1, 3%). The overall neuropsychiatric events between patients with NI and with ID were not significantly different.

### 3.3. Response Rate

**Figure 1:**
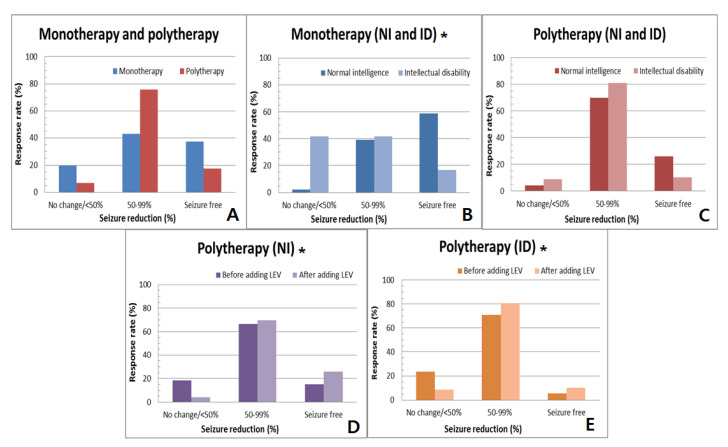
Efficacy after 6 months receiving LEV described in (**A**–**C**). (**A**) Comparing the efficacy of monotherapy and polytherapy, there was no significant difference in seizure reduction between two groups. (**B**) Comparing the efficacy of patients with NI and ID in monotherapy, patients with NI showed higher response rate of being seizure-free than those with ID (Chi-square test, *p =* 0.011). (**C**) Comparing the efficacy of patients with NI and ID in polytherapy, there was no significant difference of response rates between two groups. (**D**,**E**) Response rate of seizure reduction before and after addition of LEV in polytherapy. Both NI and ID groups were significantly affected by the addition of LEV (paired *t*-test, *p* = 0.033, *p* = 0.041, respectively). An asterisk indicates the statistically significant difference between the two groups; LEV, levetiracetam; NI, normal intelligence; ID, intellectual disability.

The number of individuals seizure-free within 6 months among the patients with NI (*n* = 55, 37%) was significantly higher than in those with ID (*n* = 18, 12%) (Table 1, *p =* 0.031). Of the 147 patients with NI, a 50–99% reduction in seizure frequency was observed in 59% (*n* = 87), and no change or <50% reduction in seizure frequency was observed in 4% (*n* = 5), while 71% of patients with ID showed a 50–99% reduction in seizure frequency (*n* = 108), leaving 17% as no change or <50% reduction in seizure frequency (*n* = 25). There was no significant difference between patients in monotherapy or polytherapy (*p =* 0.271) (Figure 1A).

Among those who received LEV as a monotherapy, patients with NI (*n* = 30, 59%) were more likely to be seizure-free than patients with ID (*n* = 6, 17%) (Figure 1B, *p =* 0.011) In polytherapy, the addition of LEV was significantly effective compared to before the addition of LEV in both groups of children (*p =* 0.033, Figure 1D and *p =* 0.041, Figure 1E, respectively). However, there was no significant difference in response rates between NI and ID who received LEV as a polytherapy (Figure 1C, *p =* 0.411). Thus, LEV was more effective in patients with NI, especially those who received LEV as a single therapy.

### 3.4. Retention Rate

**Figure 2:**
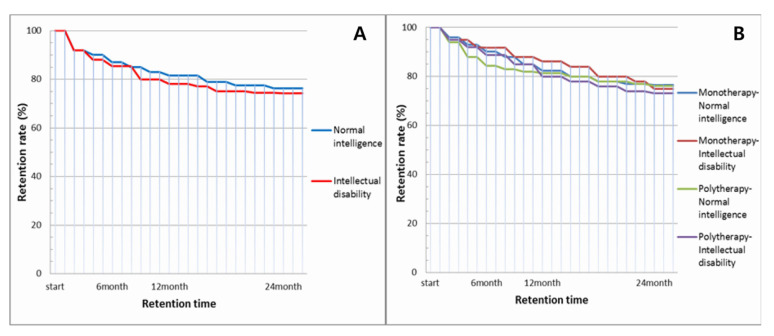
Retention rates at 6, 12, and 24 months after treatment with levetiracetam. (**A**) Patients with normal intelligence and intellectual disability. (*p =* 0.612). (**B**) Patients with normal intelligence and intellectual disability in monotherapy and polytherapy (*p* = 0.511).

The retention rates for LEV treatment after 6 and 12 months were 87% and 81%, respectively, in patients with NI, and 84% and 78%, respectively, in patients with ID (Figure 2A). Patients with NI and ID remained on LEV treatment at similar rates after 24 months (76% and 74%, respectively; *p* = 0.612). Thirty-five patients with NI and 40 patients with ID dropped out of the treatment. The most common reasons for discontinuation of LEV treatment in patients with NI and ID were lack of efficacy (11% and 13%, respectively), followed by intolerable adverse reactions (7% and 8%, respectively). Although there was a trend that patients with NI in the monotherapy group and patients with ID in the polytherapy group showed longer retention times, no statistically significant differences were observed (monotherapy: 76% with NI and 75% with ID; polytherapy: 76% with NI and 73% with ID; Figure 2B, *p* = 0511).

## 4. Discussion

ID and epilepsy appear to share common pathogenic mechanisms [9]. The prevalence of epilepsy in patients with ID is about 22% and increases with the severity of ID [10]. Therefore, it is important to ascertain the optimal ASM for pediatric patients to prevent avoidable impairment, especially in this vulnerable group. Children with neurodevelopmental disorders have higher recurrence rates after a single unprovoked seizure than those with NI [1]. In addition, prolonged or repetitive seizures and status epilepticus are more common in children with multiple disabilities and are difficult to treat [11]. Vice versa, clusters of seizures can increase the severity of the neurodevelopmental disability. Forsgren’s study reported that children with ID tend to have refractory seizures and are more likely to need polytherapy [12]. Likewise, in our study, 76% of patients with ID required treatment with more than one ASM, a higher percentage than among patients with NI (65%), suggesting that children with ID are more likely to develop ASM resistance.

Behavioral and cognitive problems can significantly impact the quality of life of children with epilepsy and can be obstacles to ASM tolerability [13]. However, certain ASMs have negative effects on cognition and bring out behavioral problems. For example, cognitive impairment can be caused by long-term use of phenobarbital, and somnolence can be caused by phenytoin [14]. Therefore, these adverse reactions to ASMs must be considered in treatment decision-making, especially for children with neurodevelopmental disorders and children who need to acquire new academic skills [15].

Adverse behavioral effects in children associated with LEV treatment affect 15-38.5% of patients with NI and more in patients with ID [16,17,18,19,20,21]. The rate of adverse reactions in this study was 14% and 10% in groups of NI and ID, respectively. As opposed to previous reports, these adverse reaction rates could be contributed to some minor side effects, including dizziness and mild fatigue, which might not have been noted in children with ID due to communication problems. Somnolence and fatigue, the most common side effects in both groups in this study, were considered minor and manageable effects and rarely led to discontinuation of LEV treatment. Neuropsychiatric adverse events, mostly irritability, hyperactivity, and aggression, were the second most frequent adverse effects in both groups, and all of these patients discontinued LEV treatment. However, children who had to discontinue LEV due to those effects comprised less than 8% of each group.

ASM that affects neurocognitive function should be avoided in school-aged children engaging in learning and skill acquisition. Although Levisohn et al. demonstrated through neurocognitive testing in children that memory and attention in children who received LEV were not significantly different from those in children who did not [6], another study showed that cognitive functions including attention, executive ability, and abstract and directional score improved after treatment with LEV [22]. Likewise, the International League Against Epilepsy (ILAE) reported that LEV could positively affect mood and cognitive functions [23]. We also have not found a noticeable decline of cognition in association with LEV treatment, and we anticipate that LEV would have less significant impacts on existing cognitive function, but additional studies related to cognitive functions are needed to confirm our hypothesis

Previous reports have suggested that even for refractory epilepsy patients, LEV had a favorable outcome and tolerable side effects [24]. LEV as an add-on treatment in pediatric patients has shown over a 50% seizure reduction in 20–60% of patients [4,25], and 20–46% of patients achieved seizure-freedom in three prospective open-label studies [26,27,28]. Although Brodtkorb, E. et al. found no significant difference in the efficacy of LEV between patients with or without ID [24], the seizure-freedom in patients with ID was lower than in the general population in some studies [29]. In our study, LEV appeared to be more effective in patients with NI since the response rate for seizure-free was greater in these patients (37%), whereas among those with ID, only 12% became seizure-free (*p =* 0.031). In a similar vein, more patients with ID (17%) had no change or a <50% reduction in seizure frequency than those with NI (4%).

Our results indicate that the tolerability of LEV is generally good, constituting about 75% of retention rates at 2 years in both groups and only minor side effects. The retention rates of patients with NI and ID were 76% and 74%, respectively, higher than other studies reporting retention rates of 47–72% [30]. This discrepancy might result from differences in the patient populations investigated. Throughout the retention time, side effects accounted for only a small portion of the reasons for the discontinuation of LEV in both groups. Thus, LEV can be a well-tolerated and safe ASM option in school-aged childhood epilepsy with ID. Since genetic alternations play an important role in epileptogenesis and neurogenesis [31], genetic testing is becoming increasingly crucial in patients with poor seizure control, other neurodevelopmental or neurological symptoms, or a strong family history of epilepsy [32] for a genetic epilepsy diagnosis. For instance, individuals with a clinical diagnosis of Dravet syndrome are likely to have a variant in a gene called *SCN1A,* and sodium-channel blockers should be avoided for treatment. Although several studies demonstrated that LEV showed effectiveness on epilepsy with genetic alteration (e.g., *CACNA1* and *STXBP1* mutation) [33,34], studies based on the efficacy of LEV as a treatment for epilepsy with genetic alterations are very scarce. Only a small number of children underwent genetic tests in this study; therefore, further extensive research efforts are required to identify the therapeutic effect of LEV associated with genetic abnormalities. The main limitations of this study were its retrospective collection of data from medical charts, small sample of pediatric patients, and short length of follow-up observations during LEV treatment. Further prospective studies using larger cohorts are needed to establish a standard treatment protocol for children with epilepsy and ID. Since our results of the response rate to LEV were assessed as three categorical values, we consider that these categorical values might not represent the actual meaning of original continuous values and might have a risk of reducing statistical power. To instill confidence in the use of LEV, well-designed, double-blind, randomized controlled trials are needed to estimate the efficacy, adverse reactions, and tolerability of LEV in children with epilepsy and ID.

## 5. Conclusions

In pediatric epilepsy with ID, especially in school-age children, choosing the appropriate ASM is an unavoidable and important factor to protect against deterioration of neurodevelopment. The goal of ASM in children with epilepsy and ID is complete control of seizures and a positive impact on cognitive function without side effects. In this study, about 83% of children with ID showed ≥50% of seizure reduction, and 74% of them showed 2 years of retention rate without serious adverse reactions, confirming that LEV could be effective, well-maintained, and safe not only for the general population but also for such vulnerable children. However, the limited information derived due to the small sample included in this study suggests that more data on the effectiveness and practical utility of LEV based on controlled studies among children with ID and epilepsy are needed.

## Figures and Tables

**Table 1:** Demographic and clinical characteristics.

Variables	Normal Intelligence *n* = 147 (49%)	Intellectual Disability *n* = 151 (51%)	*p* Value
Gender, *n* (%)			0.918
Female: Male	70 (48%): 77 (52%)	71 (47%): 80 (53%)	
Mean age, years (±SD)	12.3 (±4.6);	12.0 (±4.7);	0.422
Mean age at seizure onset, years (±SD)	5.5 (±4.6)	4.5 (±4.3)	0.232
Seizure type, *n* (%)			0.390
Localized	28 (19%)	17 (11%)	
Generalized	83 (56%)	81 (54%)	
Mixed/Unclassified	36 (25%)	53 (35%)	
Etiology of epilepsy, *n* (%)			0.074
Cerebral infection	4 (2.5%)	4 (2.5%)	
Perinatal problems	7 (5%)	30 (20%)	
Structural malformation	6 (4%)	43 (29%)	
Metabolic abnormality	3 (2%)	5 (3.5%)	
Vascular abnormality	6 (4%)	8 (5%)	
Tumor	4 (2.5%)	4 (2.5%)	
Trauma	3 (2%)	5 (3.5%)	
Genetic	10 (7%)	12 (8%)	
Idiopathic/Unknown	104 (71%)	40 (26%)	
Brain MRI, *n* (%)			0.044
Normal	101 (69%)	47 (31%)	
Abnormal	44 (30%)	99 (66%)	
Missing data	2 (1%)	5 (3%)	
Mean maximal dosage of LEV (mg/kg/day)	33.0	33.5	0.517
Monotherapy, *n* (%)	51 (35%)	36 (24%)	0.047
Polytherapy, *n* (%)	96 (65%)	115 (76%)	
Number of concomitant ASMs			
1	68	50	
2	21	36	
≥3	7	29	

MRI, magnetic resonance image; LEV, levetiracetam; ASM, anti-seizure medication.

**Table 2:** Efficacy, safety, and tolerability of levetiracetam.

Variables	Normal Intelligence (*n* = 147)	Intellectual Disability (*n* = 151)	*p* Value
Response to LEV ^(a)^			0.031
No change or <50%	5 (4%)	25 (17%)	
50–99%	87 (59%)	108 (71%)	
Seizure-free	55 (37%)	18 (12%)	
Adverse event			0.397
No	127 (86%)	136 (90%)	
Yes	20 (14%)	15 (10%)	
Retention rate (%)			0.612
<6 months	128 (87%)	129 (84%)	
12 months	120 (81%)	118 (78%)	
>24 months	112 (76%)	111 (74%)	

LEV, levetiracetam; ^(a)^ based on the reduction in seizure frequency (%).

## Data Availability

The data presented in this study are available on request from the corresponding author. The data are not publicly available due to national regulations.
